# A Case Report on Renal Abscess With Subsequent Perinephric Extension in a Patient With Poorly Controlled Type 2 Diabetes Mellitus

**DOI:** 10.7759/cureus.106961

**Published:** 2026-04-13

**Authors:** Alaa A Elhadi, Farah A Abu Assi, Tala N Almaliti, Laelas Barnia, Hala Alsabea, Nada A Abouhelwo, Ammar Alkhalili

**Affiliations:** 1 Clinical Sciences, College of Medicine, University of Sharjah, Sharjah, ARE; 2 Urology Department, Sheikh Khalifa General Hospital, Umm Al Quwain, ARE

**Keywords:** complicated pyelonephritis, klebsiella pneumonia, percutaneous drainage, perinephric abscess, renal abscess, urinary tract infection

## Abstract

Renal and perinephric abscesses are rare but potentially life-threatening complications of urinary tract infections (UTIs), especially in patients with diabetes mellitus. Diagnosis is often overlooked and delayed due to nonspecific clinical features and incomplete response to antimicrobial therapy. We report a case of a 62-year-old female with a history of poorly controlled type 2 diabetes mellitus (T2DM) who presented with flank pain, dysuria, vomiting, and fever. Imaging with contrast-enhanced computed tomography of the kidneys, ureters, and bladder (CT KUB) revealed right-sided acute pyelonephritis with an abscess in the mid-pole measuring over 5 cm, extending into the perinephric space. In spite of initial intravenous (IV) antibiotics, persistent clinical symptoms and elevated inflammatory markers required broad-spectrum antibiotics and image-guided percutaneous drainage. Culture of the abscess grew *Klebsiella Pneumoniae*, consistent with a urinary source of infection. Following drainage and targeted antibiotic therapy, the patient showed significant improvement in symptoms and inflammatory parameters, with complete recovery on follow-up. This case emphasizes the significance of early imaging, rapid emphasis on antibiotic use, and punctual percutaneous drainage in diabetic patients with recurrent UTIs to prevent septic complications and long-term renal damage.

## Introduction

A renal abscess is a circumscribed, encapsulated accumulation of pus and necrotic debris arising from bacterial destruction of renal parenchyma. It may be confined to the intrarenal tissue or extend into the adjacent perinephric space while remaining a localized infectious focus [1-3]. This pattern of spread is influenced by Gerota’s fascia, a connective tissue layer enclosing the kidney and surrounding fat, which initially limits the infection but allows extension into the perinephric space once disrupted.

Renal abscesses are relatively rare, accounting for less than 1% of all intra-abdominal abscesses. The annual incidence of renal abscesses ranges from approximately 1 to 10 cases per 10,000 individuals [2]. Renal abscesses arise through multifactorial pathological processes, most commonly resulting from ascending UTIs or, less commonly, hematogenous bacterial dissemination. Renal abscess formation is strongly associated with comorbidities such as diabetes mellitus, immunosuppression, urinary tract obstruction, genitourinary anatomical anomalies, recurrent or complicated UTIs, and pregnancy [2,3].

Renal abscesses usually develop gradually, typically presenting with fever and flank pain, and are often accompanied by chills or vague abdominal discomfort. On examination, costovertebral angle tenderness and guarding of the upper lumbar or abdominal muscles [3,4]. In this case report, we present a rare case of a renal parenchymal abscess that progressed to a perinephric abscess in a 62-year-old diabetic female following a urinary tract infection and pyelonephritis. The clinical relevance of this case lies in the presence of diabetes mellitus as a significant predisposing factor for severe renal infection, as well as the successful management of an extensive abscess using a minimally invasive approach, highlighting its role as an effective alternative to more invasive surgical interventions.

## Case presentation

History and physical examination

A 62-year-old woman with a known history of T2DM and hypertension presented to the emergency department with right-sided flank pain of three days’ duration, associated with fever, repeated vomiting, dysuria, poor appetite, and generalized weakness. She denied abdominal distension, constipation, hematuria, recent trauma, chest pain, shortness of breath, or recent hospitalization. The patient denied recent antibiotic use or prior recurrent urinary tract infections. On examination, the patient was alert and oriented. Her vitals revealed a temperature of 37.0 °C, blood pressure of 144/78 mmHg, and were otherwise unremarkable. General examination showed no acute distress. Conjunctivae were normal with reactive pupils. The neck was supple, the trachea was midline, and there was no cervical lymphadenopathy.

Abdominal examination revealed right flank tenderness with positive costovertebral angle tenderness on the right side, without abdominal distension or peritoneal signs.

Investigations

Initial laboratory evaluation revealed leukocytosis, anemia, elevated inflammatory markers, hyperglycemia, elevated serum ketones, and mild hyponatremia. Urinalysis showed significant pyuria, glycosuria, and ketonuria (Table 1). Venous blood gas analysis did not support diabetic ketoacidosis; it was consistent with stress hyperglycemia with ketosis rather than diabetic ketoacidosis (Table 1). High Hb1Ac reading confirmed uncontrolled diabetes status. Blood cultures were negative. Candida species were identified in the urine culture; however, this finding was most likely attributable to contamination from a non-clean-catch urine sample rather than true infection, while wound culture from the renal abscess later identified Klebsiella pneumoniae, sensitive to most antibiotics tested, including amikacin, ceftriaxone, ciprofloxacin, and meropenem. Renal function was preserved at admission (Table 1). 

**Table 1:** Select laboratory investigations on admission

Test	Result	Reference range
Blood tests		
White blood cell count (WBC, ×10⁹/L)	12.8	4.0-10.0
Hemoglobin (Hgb, g/dL)	10.7	12.0-15.0
C-reactive protein (CRP, mg/L)	302.6	<3.0
Procalcitonin (ng/mL)	1.53	<0.10
Serum sodium (mmol/L)	131.0	135.0-145.0
Random blood glucose (mmol/L)	20.8	3.9-11.1
Hemoglobin A1c (%)	14.2	Normal: <5.7, prediabetes: 5.7-6.4, diabetes: ≥6.5, target for diabetics: <7
Serum ketone (mmol/L)	2.8	<0.6
Serum creatinine (µmol/L)	58.0	45.0-90.0
Estimated GFR (mL/min/1.73m2)	99.3	≥90.0
Blood urea nitrogen (mmol/L)	9.9	2.5-7.1
Venous blood gases		
pH	7.49	7.35-7.45
Bicarbonate (𝐻𝐶𝑂3, mmol/L)	26.3	22.0-28.0
Lactate (mmol/L)	1.0	0.5-2.0
Urinalysis (UA)		
UA glucose	++++	Negative
UA ketone	++	Negative
UA WBCs	31-50 per HPF	0-5 per HPF

**Figure 1:**
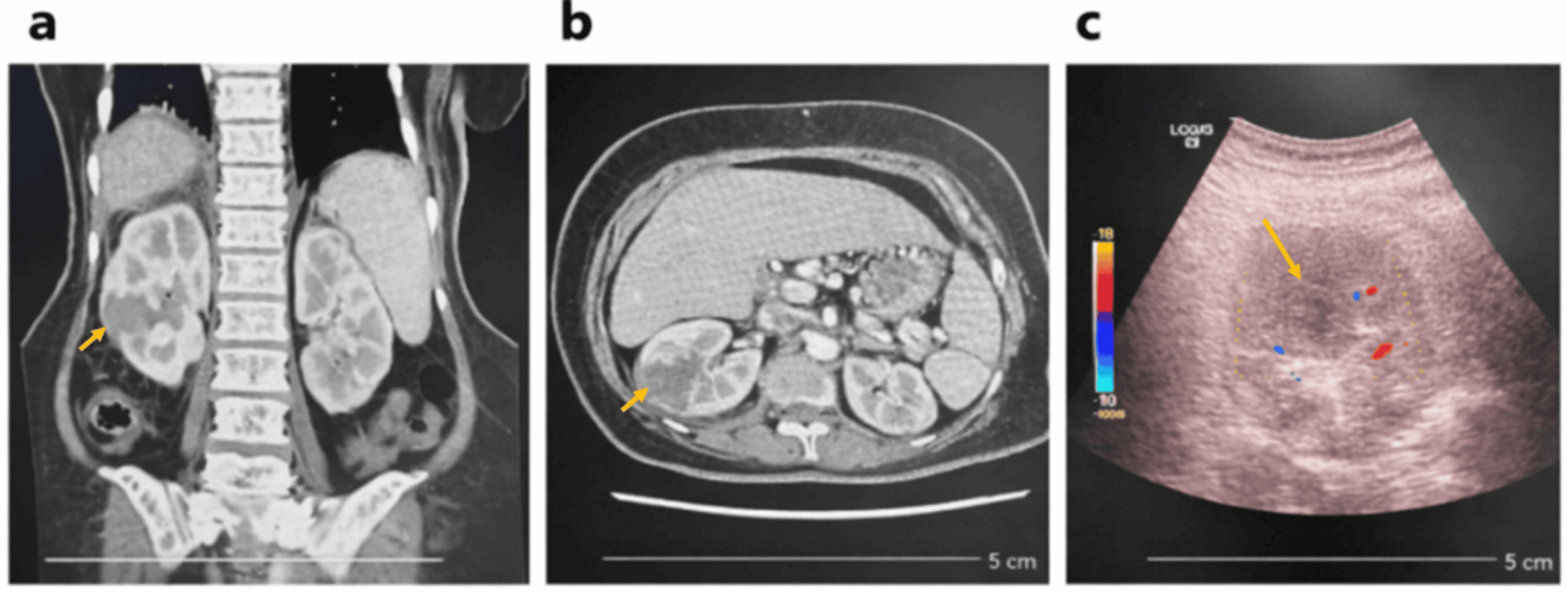
Imaging findings of the renal abscess. (A) Coronal contrast-enhanced CT image demonstrating a hypodense lesion in the kidney with features consistent with a renal abscess, associated with surrounding inflammatory changes. (B) Axial contrast-enhanced CT image showing the renal abscess with peripheral enhancement and adjacent perinephric fat stranding. (C) Grey-scale and color Doppler ultrasound image revealing a heterogeneous mixed cystic lesion with minimal internal vascularity, supporting the diagnosis of a renal abscess.

CT KUB (21/10/2025) with contrast demonstrated right-sided acute pyelonephritis with an abscess in the mid-pole measuring approximately 5.3 × 4.6 × 2.8 cm, extending into the perinephric space (Figure 1a, 1b). There was associated perinephric stranding but no evidence of renal calculi or hydronephrosis. No gas was identified within the renal parenchyma, abscess cavity, or collecting system. The left kidney and other abdominal organs appeared normal; however, the lower lobe of the right lung showed atelectatic changes with mild effusion. A follow-up ultrasound on 24/10/2025 confirmed the presence of a heterogeneous mixed cystic lesion in the right kidney, consistent with a renal abscess with perinephric extension (Figure 1c).

Differential diagnosis

The working differentials included complicated acute pyelonephritis, emphysematous pyelonephritis, perinephric abscess, and obstructive uropathy. The absence of calculi and gas on imaging excluded obstructive and emphysematous causes. The findings of a localized hypodense collection with perinephric extension on CT confirmed the diagnosis of a right renal abscess secondary to acute pyelonephritis.

Management

The patient was initially managed with intravenous ciprofloxacin (400 mg every 12 hours) as empiric therapy for presumed complicated urinary tract infection/acute pyelonephritis, in accordance with standard practice while awaiting culture results. IV (intravenous) fluids and insulin sliding scale for stress hyperglycemia were initiated. Antihypertensive therapy with amlodipine was continued. Due to persistent fever, elevated CRP, and radiological evidence of abscess formation, her antibiotic regimen was escalated to meropenem (1 g IV every eight hours) on 22/10/2025 after urology consultation. Meropenem was administered from 22/10/2025 to 24/10/2025. Hypokalemia was corrected with IV and oral potassium supplementation. 

On 24/10/2025, percutaneous drainage of the right renal abscess was performed under combined ultrasound and fluoroscopic guidance. A 10.2 Fr Dawson-Mueller drainage catheter was inserted into the abscess cavity, and approximately 2 mL of purulent material was aspirated and sent for culture. The procedure was performed under local anesthesia using 2% lidocaine, and there were no immediate complications. The drain was connected to a collection bag, and pus drainage was noted to be free-flowing. 

Post-procedure, the patient’s clinical condition improved with a gradual reduction in pain, normalization of temperature, and declining CRP levels (from 302.6 to 39.5 mg/L by 29/10/2025). Glycemic control was optimized with basal-bolus insulin (Insulin Aspart three times a day and insulin glargine nightly). Supportive therapy included IV fluids, pantoprazole, metoclopramide for nausea, and paracetamol for analgesia. 

Outcome and follow-up

**Figure 2:**
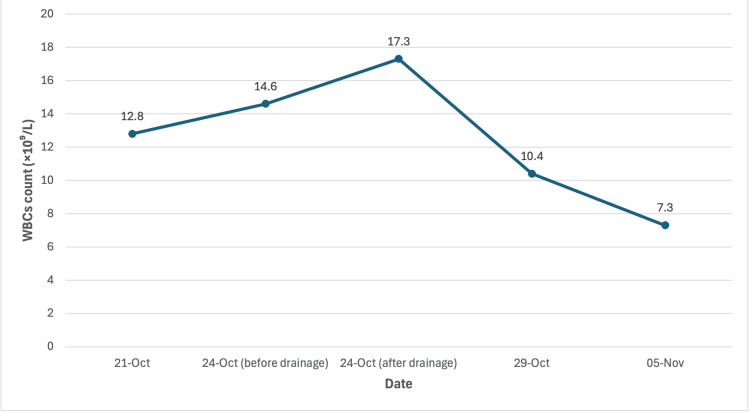
WBC trend over time WBC: white blood cell

**Figure 3:**
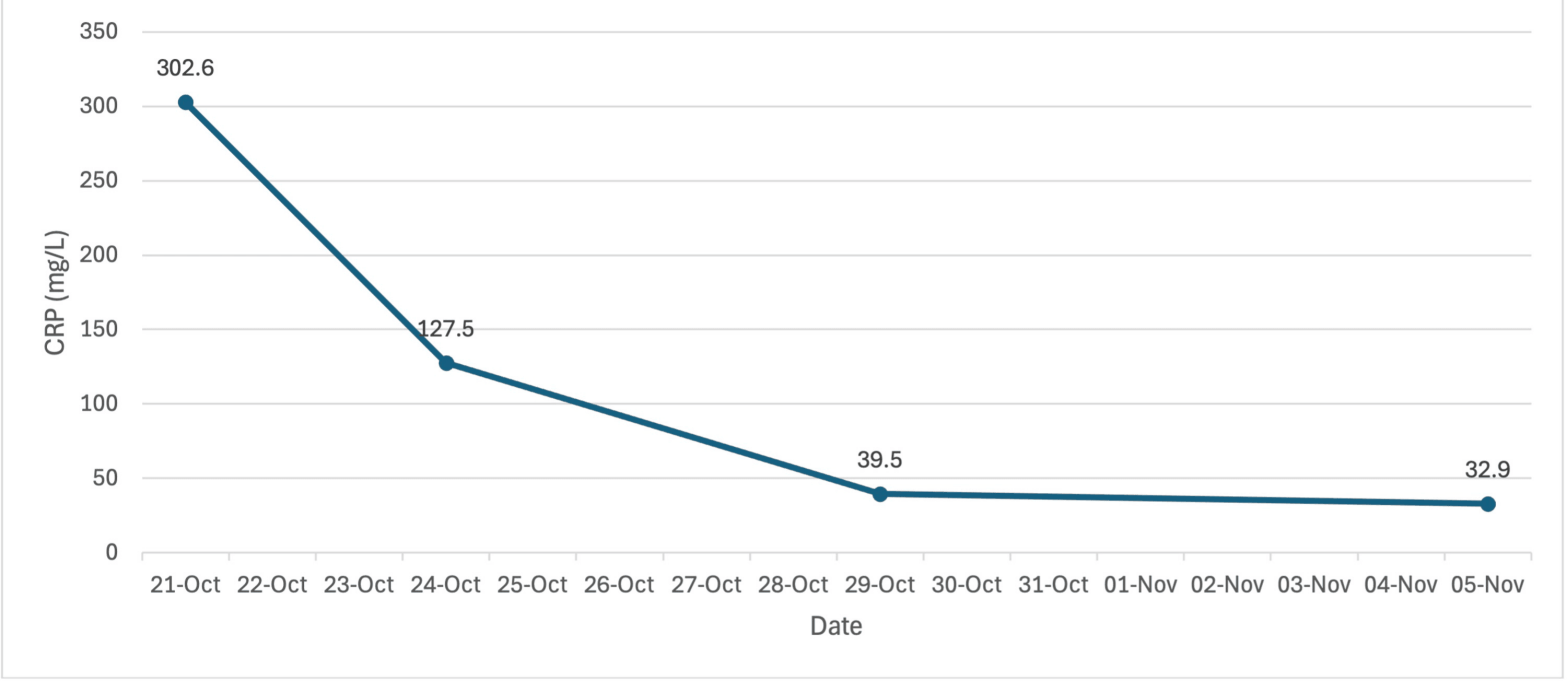
CRP trend over time CRP: C-reactive protein

Following drainage, the patient showed steady improvement in symptoms and inflammatory parameters. According to Figure 2 and Figure 3, WBC trended down to 7.3 ×10⁹/L and CRP to 32.9 mg/L by 05/11/2025. There was no recurrent fever or flank tenderness. The patient remained hemodynamically stable and afebrile, with improved glycemic control and stable renal function (creatinine 53 µmol/L, eGFR 101.5 mL/min), and the drain was removed prior to discharge. She was discharged with oral ciprofloxacin (500 mg, twice daily for two weeks) based on sensitivity and an endocrinology follow-up appointment for diabetic management. 

The patient was scheduled for follow-up with urology and endocrinology for reassessment. At two weeks of follow-up, she remained asymptomatic with normal renal parameters, and bedside ultrasound imaging was consistent with the clinical picture and showed improvement and resolution of the abscess. 

Final diagnosis

Right renal abscess secondary to Klebsiella pneumoniae infection in a patient with uncontrolled T2DM, successfully managed with percutaneous image-guided drainage and intravenous antibiotics.

## Discussion

Renal and perinephric abscesses are uncommon but potentially life-threatening complications of UTIs, most frequently arising from inadequately treated or rapidly progressive acute pyelonephritis. Several risk factors have been consistently identified, including DM, immunosuppression, obesity, prolonged urinary catheterization, and structural abnormalities of the urinary tract. Among these, DM is recognized as one of the most important determinants of both abscess formation and disease progression [2,5].

In the present case, uncontrolled T2DM was a central contributor to disease severity and clinical course. Chronic hyperglycemia impairs innate immune mechanisms, particularly neutrophil chemotaxis, phagocytosis, and intracellular killing, while simultaneously promoting bacterial growth within renal parenchyma. These factors markedly increase susceptibility to complicated UTIs and favor progression from acute pyelonephritis to renal and perinephric abscess formation. Although Hemoglobin A1c (HbA1c) was unavailable, the markedly elevated random blood glucose level and ketonemia on admission strongly suggest poor long-term glycemic control, which likely contributed to both abscess development and delayed response to antimicrobial therapy 

Microbiologically, renal abscesses most commonly involve gram-negative enteric organisms, particularly *Escherichia coli *and *Klebsiella pneumoniae*, with *Staphylococcus aureus* more frequently associated with hematogenous spread [5]. In this case, *Klebsiella pneumoniae *was isolated from the abscess drainage culture, consistent with a urinary source and in keeping with reported patterns in diabetic patients. The infection was limited to the kidney and surrounding perinephric tissue with no metastatic spread to other organs, and no further microbiological characterization (e.g., strain typing or virulence profiling) was performed. Klebsiella infections are known to be associated with more aggressive disease, higher rates of abscess formation, and increased likelihood of perinephric extension, particularly in the setting of impaired host defenses [2,6]. Notably, *Candida* species were identified in the urine culture; however, this finding was most likely attributable to contamination from a non-clean catch sample rather than true infection. This interpretation is supported by the isolation of *Klebsiella pneumoniae *from the abscess (wound) culture, confirming a bacterial etiology for the pyelonephritis. Consequently, the candiduria was considered clinically insignificant and was not treated.

Clinically, renal and perinephric abscesses often present with nonspecific and insidious symptoms, which can delay diagnosis. Typical manifestations include fever, flank or abdominal pain, vomiting, malaise, and generalized weakness, while classic lower urinary tract symptoms may be absent in a significant proportion of patients [5]. Notably, our patient presented with right-sided flank pain, fever, repeated vomiting, dysuria, and costovertebral angle tenderness, reflecting a combination of acute pyelonephritis features and early deep-seated infection. The presence of dysuria in this case may suggest rapid progression from UTI to abscess formation rather than isolated cystitis. Although pallor, weight loss, and night sweats have been described in chronic presentations, their absence in our patient is consistent with relatively early recognition of the suppurative process. Importantly, no palpable flank mass was detected, a finding reported in up to 60% of perinephric abscesses but not universally present.

Failure to improve with appropriate initial antimicrobial therapy should raise suspicion for renal or perinephric abscess. In our case, empirical intravenous ciprofloxacin was initiated for presumed acute pyelonephritis; however, persistent fever and elevated inflammatory markers prompted further evaluation. Contrast-enhanced CT imaging confirmed a renal abscess measuring over 5 cm with extension into the perinephric space, establishing the diagnosis of complicated pyelonephritis. CT remains the imaging modality of choice, as it not only demonstrates renal abscesses as hypodense, relatively well-defined lesions, but also accurately delineates the full extent of disease, including associated perinephric fat stranding and fluid collections that indicate extra-renal spread. In contrast, ultrasound typically identifies these lesions as heterogeneous hypoechoic areas with internal echoes and may demonstrate peripheral vascularity on Doppler imaging without internal flow. However, ultrasound is limited by less precise margin definition and reduced sensitivity in detecting perinephric extension, often underestimating the true extent of disease. Consequently, CT plays a crucial role in comprehensive assessment and management planning [5].

Regarding antimicrobial therapy, existing literature and expert recommendations suggest that empiric treatment for renal or perinephric abscess should include broad-spectrum gram-negative coverage, with consideration of anti-methicillin-resistant *Staphylococcus aureus *(MRSA) agents such as vancomycin, particularly in severe or healthcare-associated infections. In this patient, escalation from ciprofloxacin to meropenem provided robust coverage against resistant gram-negative organisms, including Klebsiella pneumoniae. Vancomycin was not added, which was clinically reasonable given the absence of significant risk factors for MRSA infection, negative blood cultures, and subsequent culture data confirming a gram-negative pathogen sensitive to carbapenems [2]. This targeted escalation aligns with antimicrobial stewardship principles while ensuring adequate coverage for the identified organism.

Abscess size and extension are critical determinants of definitive management. Abscesses measuring ≥5 cm, those with perinephric extension, or those associated with systemic illness are unlikely to resolve with antibiotics alone due to poor antimicrobial penetration and increased risk of spontaneous rupture. In such cases, image-guided percutaneous drainage is recommended as first-line therapy [2]. Given the size of the abscess and perinephric involvement in our patient, percutaneous drainage under ultrasound and fluoroscopic guidance was appropriately performed, resulting in prompt clinical and biochemical improvement. The transient rise in leukocyte count immediately following drainage from (14.7 x 10^3^ to 17.3 x 10^3^) likely reflected a procedural inflammatory response, followed by sustained normalization over subsequent days to (7.3 x 10^3) as shown in (Figure 2), supporting effective source control.

An additional noteworthy feature in this case was the presence of pleural effusion and atelectatic changes on imaging. Similar findings have been described in association with renal and perinephric abscesses, likely due to contiguous spread of inflammation across the diaphragm or reactive pleural involvement. In reported cases, pleural effusions associated with perinephric abscesses are often reactive rather than infectious and tend to resolve concurrently with treatment of the primary abdominal pathology [7]. In our patient, the effusion was small and clinically stable, and no thoracentesis was performed. The parallel improvement of pulmonary findings following abscess drainage supports a reactive inflammatory mechanism rather than primary pulmonary pathology.

Overall, this case underscores DM as a key driver of progression from UTI to renal and perinephric abscess, as well as a contributor to initial antimicrobial treatment failure. It highlights the importance of early imaging in diabetic patients with persistent symptoms despite appropriate antibiotics, the role of culture-directed antimicrobial escalation, and the effectiveness of timely percutaneous drainage in preventing septic deterioration and long-term renal damage

## Conclusions

This case highlights renal and perinephric abscess secondary to *Klebsiella pneumoniae *as a serious and potentially life-threatening complication of UTI in patients with poorly controlled diabetes mellitus.

Renal and perinephric abscesses should be suspected in diabetic patients who have persistent symptoms despite appropriate antibiotic therapy, particularly in the presence of ongoing fever, leukocytosis, and elevated inflammatory markers.

Early contrast-enhanced CT imaging is essential when clinical improvement is inadequate, as it facilitates timely diagnosis, appropriate escalation of antimicrobial therapy, and image-guided drainage.

Prompt source control through minimally invasive drainage, combined with targeted antimicrobial therapy, can lead to favorable outcomes even in patients with significant risk factors, as demonstrated in this case, where, despite multiple risk factors, the patient had a positive clinical outcome, highlighting the critical role of early source control in managing such infections.
